# Enhancing the Uniformity of Bowl-Shaped Gold Nanoparticles Using a Dynamic System in an Electrochemical Microfluidic Chip

**DOI:** 10.3390/nano16100640

**Published:** 2026-05-21

**Authors:** Kueakul Khowamnuaychok, Chumphon Luangchaisri, Chivarat Muangphat

**Affiliations:** 1 Department of Physics, Faculty of Science, King Mongkut’s University of Technology Thonburi, 126, Pracha Uthit Rd., Bang Mod, Thung Khru, Bangkok 10140, Thailand; 2 Materials Technology Program, School of Energy, Environment and Materials, King Mongkut’s University of Technology Thonburi, 126, Pracha Uthit Rd., Bang Mod, Thung Khru, Bangkok 10140, Thailand

**Keywords:** bowl-shaped gold nanoparticles, hydrogen nanobubbles, electrochemical synthesis, microfluidic system, plasmonic nanoparticles, surface plasmon resonance

## Abstract

Bowl-shaped gold nanoparticles (BAuNPs) are of significant interest due to their tunable localized surface plasmon resonance (LSPR) properties. This report presents a new synthesis method that uses hemispherical hydrogen nanobubbles on planar, non-conducting surfaces as templates for gold shell deposition. Initial synthesis under stagnant conditions yielded non-uniform sub-micron particles, attributed to localized hydrogen concentration gradients. The cyclonic flow was introduced aiming to reduce these gradients, although simultaneously inducing significant particle aggregation, obscuring the open structure. To overcome these challenges, an electrochemical microfluidic system was implemented to create a laminar flow environment. This configuration optimizes ion distribution and introduces shear forces that promote particle detachment, successfully limiting particle dimensions to below 200 nm, and preventing the accumulation. Systematic optimization identified optimal parameters: an ideal channel length of 7.5 mm, an applied potential of −0.6 V, and a flow rate of 0.028 µL s^−1^. These parameters that strike a balance between nanobubble growth and gold deposition kinetics can produce highly uniform BAuNPs with a well-defined open structure and thin solid gold shells, with an outer diameter of 105.3 ± 12.1 nm and a core diameter of 80.1 ± 11.9 nm. These structural parameters successfully shift the plasmonic resonance to 760 nm, which responds perfectly with the first biological window for potential in vivo biomedical applications.

## 1. Introduction

According to their non-toxicity, tunable optical properties, and superior biocompatibility [1,2], gold nanoparticles have gained significant attention in the medical industry. These characteristics facilitate a diverse range of in vitro and in vivo applications [3], including diagnostic [4,5], biosensing [6,7], bioimaging [8,9,10], and target drug delivery [11,12]. To date, research has primarily focused on diverse morphologies, ranging from solid geometries such as nanospheres, nanorods, nanodisks, and nanotriangles [13,14,15,16,17] to hollow architectures like nanotubes, hollow nanospheres, and nanocages [18,19,20,21,22,23].

Among these, open-structured or bowl-shaped gold nanoparticles (BAuNPs) remain less explored compared to solid and hollow gold nanoparticles. However, their distinct open-cavity geometry and sharp rim edges facilitate the concentration of electromagnetic fields, generating highly localized plasmonic “hotspots”. This structural asymmetry results in excellent localized surface plasmon resonance (LSPR) and surface-enhanced Raman scattering (SERS) properties compared to their solid counterparts [24,25].

The transition from symmetrical to asymmetrical core–shell structures significantly broadens the tunability of LSPR and SERS. The Halas research group [26,27] demonstrated that reshaping 100-nm dielectric-core nanoshells into nanocups and nanocaps via an electron-beam-induced ablation shifted the long-axis dipolar plasmon resonance from 690 nm to 850 nm, while simultaneously generating a secondary quadrupole resonance at 610 nm. Theoretical evaluations using the three-dimensional finite-difference time-domain (FDTD) method showed strong agreement with these experimental results, suggesting that nanocups are well-suited for in vivo applications.

Similarly, Ye et al. [28] synthesized individual gold semi-shells with silica (SiO_2_) cores via ion milling, then etched the cores to produce gold nanobowls. Both the semi-shell and nanobowl structures exhibited comparable SERS properties at 633 nm, driven by plasmonic coupling and symmetry reduction, as predicted by FDTD simulations. Furthermore, ordered arrays of gold nanobowls on solid substrates, fabricated through colloidal crystal templating, exhibit strong SERS enhancement due to their concave geometry and plasmonic coupling between neighboring bowls [29,30]. Wi, Son, and Lee [31] utilized nanoimprint lithography to develop a uniform array of 200-nm-diameter gold nanobowls as plasmonic sensing platforms for mesoscale analytes, including viruses and exosomes. Their findings indicated that the resonance wavelength of the bowl-shaped structure shifts by 40 nm, from 960 nm to 1000 nm, upon analyte interaction, whereas a 2-dimensional gold disk shifts from 940 nm to 944 nm. Furthermore, the LSPR peak exhibited a concentration-dependent shift, reaching 1175 nm at a bead concentration of 3.6 nM. Despite these advantages, a significant drawback of using a large-area nanobowl array on a substrate is its limited application in solution-based sensing environments compared to individual colloidal nanoparticles.

Recent investigations into colloidal BAuNPs emphasize their superior performance in sensing and catalysis. Kasztelan et al. [32] noted that the plasmonic activity localized at the edges of 30–40 nm nanobowls provided significantly higher SERS enhancement factors for the detection of rhodamine 6G (R6G) compared to spherical or urchin-shaped particles. The results revealed that the bowl-shaped structures exhibited a significantly higher enhancement factor compared to the spherical and urchin shapes. This highlighted how the complex asymmetry of colloidal BAuNPs manipulates optical properties, fostering the development of new classes of nanoparticles suitable for plasmonic sensing and optical investigations.

Beyond optics, the high surface-area-to-volume ratio and open-shell structure of BAuNPs improve catalytic performance by overcoming diffusion-limited mass transport. Building on this advantage, Pedireddy et al. [33] leveraged it to synthesize nanoporous gold bowls composed of interconnected sub-10 nm ligaments. This 3D network improved the electrocatalytic activity for methanol oxidation by nine times that of 8 nm solid gold nanoparticles and three times that of conventional 2D nanoporous sheets. This enhanced performance is attributed to the open-shell structure, lattice strain, and increased electroactive surface area.

The efficacy of bowl-shaped geometries extends to other metals as well. For example, Zhang et al. [34] successfully synthesized hollow, porous platinum (Pt) bowls with a diameter of 300 nm via a one-pot hydrothermal method, demonstrating a mass activity approximately 3.1 times higher than that of solid Pt spheres. Similarly, Bera et al. [35] investigated the effect of bowl-shaped phosphate-based nanoparticles, achieving a palladium (Pd) loading capacity of 36.46%, whereas spherical carriers achieved only 12.46%. This higher loading led to vastly superior performance in catalytic carbon–carbon (C–C) coupling reactions. It was observed that the bowl-shaped structures exhibited impressive plasmonic properties with a large surface area, thereby enhancing their chemical activity.

The critical limitations of current colloidal nanobowls synthesized via ion milling or lithography include the retention of a dielectric core, a diameter exceeding 200 nm, and a lack of individuality in liquid phases. These factors significantly reduce the active surface area and limit their suitability for in vivo applications, due to challenges in transport and clearance processes across various organs and tissues [36]. Consequently, there is a critical requirement for a synthesis strategy that produces small, core-free, and highly uniform colloidal BAuNPs capable of strong optical absorption within the biological window (650–900 nm) [8].

This work presents the first integration of electrochemical and microfluidic systems for the one-step synthesis of individual, uniform, and core-free BAuNPs with a diameter below 200 nm. Advancements in this strategy overcome the limitations of traditional methods, maximizing the potential of these nanostructures for advanced optical and biomedical applications. Furthermore, this electrochemical microfluidic approach establishes a novel, scalable framework with significant potential for producing BAuNPs at high volume.

## 2. Materials and Methods

### 2.1. Materials

All chemicals and analytical-grade (AR) reagents were used as received without further purification. The primary gold precursor, sodium gold sulfite (Na_3_Au(SO_3_)_2_), was purchased from Technic Inc., Cranston, RI, USA. Supporting chemical agents, including ethylenediamine (C_2_H_4_(NH_2_)_2_) and nickel sulfamate (Ni(SO_3_NH_2_)_2_·4H_2_O), were obtained from Sigma-Aldrich, St. Louis, MO, USA, while sulfuric acid (H_2_SO_4_, 95–97 wt%) was purchased from Merck, Darmstadt, Germany. Lithographic and microfluidic fabrication materials were acquired from several specialized suppliers: AZ 1512 HS positive photoresist and AZ 400K developer (1:4) were sourced from MicroChemicals GmbH, Ulm, Germany. Dow Corning, Midland, MI, USA, provided Sylgard 184 Polydimethylsiloxane (PDMS) elastomer kit, and sodium carbonate (Na_2_CO_3_, 1.5 wt%) was sourced from Flinn Scientific, Inc, Batavia, IL, USA. Substrate preparation utilized commercial epoxy-copper-clad printed circuit boards (PCBs) from Electronics Design Laboratory, Ltd., Bangkok, Thailand, alongside Riston Special FX Series (FX515) dry negative photoresist film from DuPont de Nemours, Inc., Wilmington, DE, USA, and commercial copper etchant from Future Kit, Bangkok, Thailand.

Electrochemical components, specifically the platinum mesh counter electrode and an Ag/AgCl reference electrode, were acquired from Ted Pella, Redding, CA, USA, and BASi Science, West Lafayette, IN, USA. Finally, analytical solvents, including 2-propanol (99.7 wt%), hexane (99 wt%), and acetone (99 wt%), were supplied by Qrec, Auckland, New Zealand.

### 2.2. Characterization Techniques

The morphology and structural properties of the synthesized nanoparticles were characterized using high-resolution transmission electron microscopy (HRTEM) and scanning transmission electron microscopy (STEM) on a Talos instrument from Thermo Fisher Scientific, Waltham, MA, USA, operated at an accelerating voltage of 200 kV. Surface morphology and the spatial distribution of the nanoparticles on the substrate were examined using a Nova NanoSEM 450 field-emission scanning electron microscope (FE-SEM) from FEI (Thermo Fisher Scientific, Waltham, MA, USA). Quantitative analysis of particle size and distribution was performed on the resulting TEM micrographs using ImageJ software version 1.54i [37]. The optical characteristics were evaluated by measuring absorbance spectra across the 400–1000 nm range with a UV–Vis spectrophotometer (Halo VIS-20, Dynamica Scientific Ltd., Livingston, UK). To determine particle concentration, dynamic light scattering (DLS) was performed using a Malvern Zetasizer Ultra (Model ZSU5700, Malvern Panalytical, Malvern, UK) equipped with ZS Xplorer version 4.0.0.683 concentration analysis software. Finally, the total yield of the BAuNPs was estimated by integrating the measured particle concentration with a geometric approximation of the nanoparticle volume and geometry.

### 2.3. Fabrication Process

#### 2.3.1. Copper Stripe-Patterned Electrodes on a Polyimide Sheet

**Figure 1:**
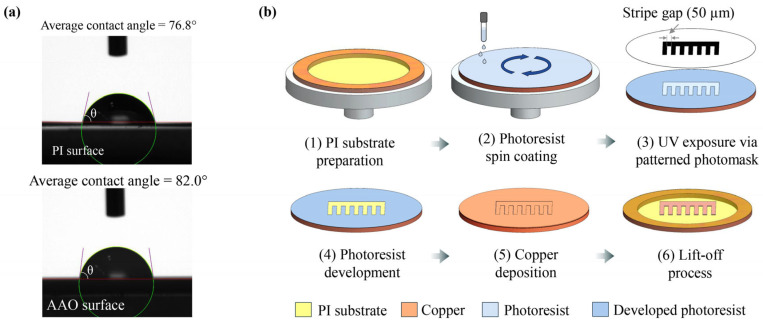
(**a**) Comparative contact angle measurements characterizing the surface wettability of PI and AAO surfaces. The indicated red baseline, purple tangent lines, and green droplet fitting contour for the contact angle (θ) analysis. (**b**) Schematic representation of the fabrication workflow for copper stripe-patterned electrodes on a PI substrate, utilizing UV photolithography and thermal evaporation.

To investigate the formation of BAuNPs on planar surfaces, the alternating stripe-patterned copper electrodes were fabricated on a non-conductive polyimide (PI) substrate. The copper stripes serve as working electrodes to generate and maintain hydrogen supersaturation, while the non-conducting regions facilitate the heterogeneous nucleation and growth of hydrogen nanobubbles. Copper was specifically selected for the working electrode due to its relatively low activity in the hydrogen evolution reaction (HER) compared to other metals [38,39,40]. This lower activity minimizes nanobubble nucleation directly on the electrode surface, thereby promoting bubble nucleation and growth at non-conductive sites. The PI surface was chosen for these nucleation sites due to its smooth morphology and inherent hydrophilicity, with a contact angle of approximately 76.8°. This provides a nucleation environment comparable to that of the conventional anodic aluminum oxide (AAO) membrane, which has a contact angle of approximately 82.0°, as depicted in Figure 1a. The surface of the AAO membrane successfully served as nucleation sites for the synthesis of hollow gold nanoparticles (HAuNPs), as suggested by Huang et al. [19,20,41].

The fabrication process, as illustrated in Figure 1b, began by stretching a 50 µm-thick PI sheet (DuPont de Nemours, Inc., Wilmington, DE, USA) over a copper ring with an inner diameter of 70 mm to ensure a perfectly planar substrate. A 2 µm-thick layer of AZ 1512 HS positive photoresist was deposited on the stretched PI sheet via spin-coating (500 rpm for 10 s, followed by 3000 rpm for 30 s) and soft-baked at 90 °C for 1 min to remove residual solvent. The substrate was then aligned with a photolithographic mask comprising 50 µm-wide dark stripes separated by 50 µm gaps, interconnected by a 2.5 mm-wide vertical dark section at one end, and exposed to UV light using an EVG 610 Mask aligner for 10 s. After exposure, the uncured areas of the photoresist were removed using a 1:4 dilution of AZ 400K Developer in deionized (DI) water for 90 s.

Subsequently, the patterned substrate was coated with a 35 nm-thick titanium adhesion layer, followed by a 35 nm-thick copper layer, using a custom-built thermal evaporation system. Finally, a lift-off process was performed by immersing the substrate in acetone for 35 s to remove any remaining photoresist, leaving the copper stripe pattern on the PI substrate. The finalized electrodes were rinsed with DI water and stored in a desiccator prior to subsequent experimental stages.

#### 2.3.2. Electrochemical Microfluidic Chip (E-MFC)

Microfluidic chips are highly integrated microscale devices engineered to manipulate small volumes of fluid through confined microchannels. By offering a high surface-to-volume ratio, these devices enhance electrochemical reaction kinetics and improve chemical reaction efficiency within a microscale environment. Due to its precision, highly flexible design, minimal waste production, and time- and cost-effectiveness, microfluidic technology has received significant attention as a compelling alternative to conventional experimental methodologies [42]. These chips can operate independently or be integrated with other miniaturized devices and detectors, such as microreactors [43], microscopes [44], and sensors [45], forming what are collectively known as lab-on-a-chip (LoC) devices. In this study, the microfluidic chip was integrated into the electrochemical system to synthesize individual BAuNPs with sizes below 200 nm by precisely controlling mass transport. The electrochemical microfluidic module comprises three main components: (1) a stripe-patterned conductive layer to serve as the electrode array, (2) a planar, non-conductive surface to facilitate hydrogen nanobubble nucleation, and (3) a microchannel to contain the solution and define the fluidic pathway.

The substrate for the electrochemical microfluidic chips (E-MFCs) was a commercial FR4 PCB because it provides a 40 µm-thick single-sided copper layer, eliminating the need for a high-vacuum facility to deposit a conductive layer. The copper layer was patterned into three parallel stripes to facilitate a two-electrode electrochemical system, where the middle stripe functions as a cathode and the two outer stripes serve as anodes. The PCB was prepared into 2.5 cm × 4 cm substrates and sequentially cleaned with metal polishing liquid, detergent, DI water, and acetone to remove surface contaminants and the native copper oxide layer.

**Figure 2:**
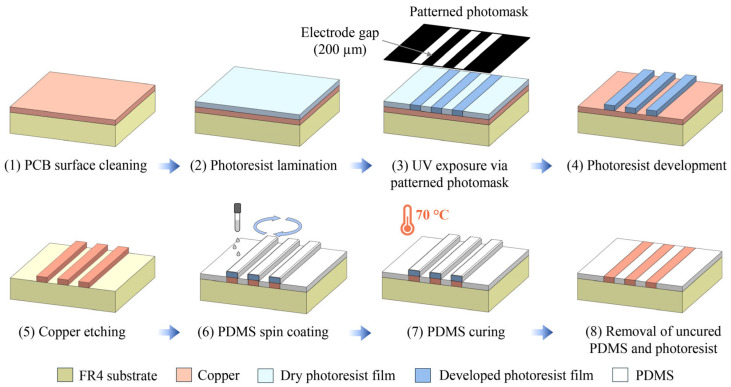
Cross-sectional schematic detailing of the fabrication process of E-MFC on a PCB substrate using UV photolithography.

As illustrated in Figure 2, a Riston FX515 dry negative photoresist film was laminated onto the substrate at 120 °C using a laminator (A3-330C Laminator, SCHLONGEN, China) to ensure robust adhesion. A dark-field photolithographic mask, featuring three parallel 200 μm-wide transparent stripes separated by 200 μm gaps, was printed by a high-resolution imagesetter (Tanto 5120, Dainippon Screen Mfg. Co., Ltd., Kyoto, Japan) on a transparent film (HLE imagesetter film, Ferrania, Italy).

The photolithography mask was placed in direct contact with the photoresist layer, and the assembly was exposed to UV–C light (8 W × 6 lamps, Philips, China) at a distance of 8 cm for 3 min inside a dark box for a controlled environment. During UV–C exposure, the transparent regions permitted UV–C to penetrate, inducing crosslinking in the photoresist to form an insoluble protective layer, while the opaque regions blocked incoming UV–C light. Following exposure, the exposed photoresist region became crosslinked, forming a durable, insoluble protective layer, while the unexposed regions were later removed during development. Following UV–C exposure, the uncrosslinked photoresist was developed in 1.5 wt% Na_2_CO_3_ and rinsed with DI water and acetone. The copper regions not protected by the cured photoresist were removed by immersion in a commercial copper etchant for 10 min, leaving the finalized three-stripe electrode configuration.

After removal of the copper layer, the underlying FR4 epoxy resin exhibits significant surface roughness due to its woven fiberglass reinforcement. To create an ideal heterogeneous nucleation site for hydrogen nanobubbles, a smoothing procedure was implemented. A PDMS solution (10:1 base-to-curing agent ratio, diluted in hexane at a 11:15 ratio) was spin-coated onto the chip at 4000 rpm, followed by thermal curing at 70 °C for 4.5 h to remove residual hexane and cure the PDMS layer. Notably, the PDMS layer in contact with the photoresist did not fully crosslink during this phase. This allowed for a selective acetone lift-off process that simultaneously removed the photoresist and the overlying uncured PDMS. The resulting construction consists of exposed copper electrodes separated by a smooth, non-conducting PDMS surface, which provides the necessary conditions for subsequent electrochemical experiments to produce hemispherical hydrogen nanobubble templates for gold shell deposition.

### 2.4. Experimental Setup

#### 2.4.1. A Three-Electrode Electrochemical System in a Bath

**Figure 3:**
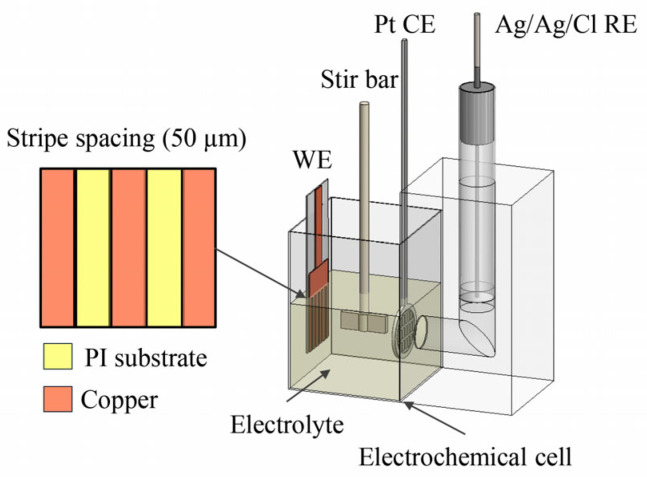
Schematic illustration of the three-electrode electrochemical setup. The stripe-patterned copper electrode functions as the working electrode (WE), complemented by a platinum mesh counter electrode (CE) and an Ag/AgCl reference electrode (RE).

The synthesis of BAuNPs on planar surfaces was initially investigated using a conventional three-electrode configuration, as illustrated in Figure 3. The experimental setup utilized the copper stripe-patterned substrate as the working electrode, a 5 mm-diameter platinum mesh as the counter electrode, and an Ag/AgCl reference electrode. These components were positioned within a rectangular polytetrafluoroethylene (PTFE) electrochemical bath with dimensions of 10 × 10 × 15 mm^3^. To minimize ohmic resistance, the working and counter electrodes were positioned in proximity, while the reference electrode was isolated in a separate channel and connected to the main bath through a 3 mm-diameter hole to maintain electrochemical contact and prevent contamination.

The experiments were conducted using a potentiostat (EG&G Princeton Applied Research Model 263A) to apply the designated potential, coupled with a data logger for real-time current monitoring. The electrolyte was prepared by mixing 2.15 mL of 0.16 M Na_3_Au(SO_3_)_2_, 0.10 mL of 7 M C_2_H_4_(NH_2_)_2_, and 0.75 mL of 0.4 M Ni(SO_3_NH_2_)_2_·4H_2_O. Following 5 min of magnetic stirring, the solution pH was precisely adjusted to 6.0 using 4 M H_2_SO_4_. The addition of Ni^2+^ ions, originating from the dissociation of nickel sulfamate, is intended to modify the electrochemical environment. Nickel has been reported to enhance hydrogen evolution kinetics due to its relatively higher exchange current density than gold [46]. In the present of nickel sulfamate, nickel ions act as the primary species depositing onto the electrodes to sustain the electrochemical current. This consumption leads to maintaining the gold concentration in the bulk solution available for deposition at the hydrogen nanobubble interface. Furthermore, the inclusion of EDA suppresses the disproportionation of the gold sulfite complex, thereby preventing unwanted precipitation and stabilizing the precursor solution throughout the synthesis [19,41]. During the synthesis process, a constant potential of −0.7 V (vs Ag/AgCl) was applied to the copper working electrode for a specific duration. After synthesis, the electrode underwent a rigorous cleaning protocol involving three sequential 1 h immersion cycles in DI water to eliminate residual electrolyte species.

Initial experiments under static conditions revealed that the level of hydrogen supersaturation, a critical parameter for nanoparticle nucleation, decreased significantly with distance from the working electrode. This spatial gradient negatively affected the size uniformity of the resulting BAuNPs, as reported in a previous study [47]. To eliminate this effect, a convective mass-transport approach was introduced using a 24 V DC motor-driven stirrer during the electrochemical process. It was hypothesized that the induced mechanical stirring would homogenize the hydrogen supersaturation gradient, thereby potentially improving the uniformity of the synthesized nanoparticles.

#### 2.4.2. A Two-Electrode Electrochemical Setup Using E-MFC

The microfluidic channel was fabricated using a 3D-printed U-shaped mold (1.4 mm wide, 20 mm long, and 0.5 mm high) on a Creality Ender-6 3D printer with ABS filament. A PDMS mixture, prepared at a 10:1 base-to-curing agent ratio, was cast into the 3D-printed mold and degassed under vacuum to remove trapped air bubbles. Following a 24 h room-temperature curing period, the U-shaped PDMS microchannel was extracted and integrated with the E-MFC substrate.

**Figure 4:**
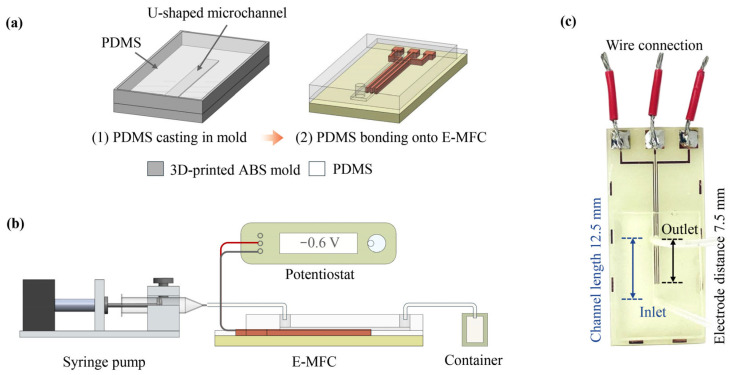
(**a**) Schematic illustration of the PDMS microchannel assembly integrated with the E-MFC. (**b**) Schematic diagram detailing the complete experimental configuration. (**c**) Photographic image of the final assembled device.

As illustrated in Figure 4, the integrated assembly was incorporated into a comprehensive electrochemical flow system. Electrical connections were established by soldering wires to the copper electrodes and interfacing them with a potentiostat for precise potential control and real-time current monitoring. Fluidic connectivity was achieved by attaching silicone tubes to supply the solution through the microchannel inlet and collect the resulting product into a collection container at the outlet. Although the total distance between the inlet and outlet is approximately 12.5 mm, the active fluidic path traverses 7.5 mm of the electrode array. The reactant solution, as mentioned in Section 2.4.1, was loaded into a 1 mL syringe and delivered via a custom-built syringe pump at a constant flow rate. To ensure system equilibrium, the solution was allowed to prime the microchannel for 5 min before initiating the electrochemical reaction.

Synthesis experiments were performed under a constant potential of −0.6 V. Upon exiting the E-MFC, the resulting colloidal suspension was collected in a centrifuge tube and immediately diluted with DI water at a 1:15 volume ratio to halt the electroless gold deposition reaction. The final BAuNP product was collected by centrifugation at 14,000 rpm for 5 min, followed by five sequential cycles of sonication-assisted washing with an IPA–DI water mixture (1:1) to ensure the complete removal of residual electrolyte species.

## 3. Results and Discussion

**Figure 5:**
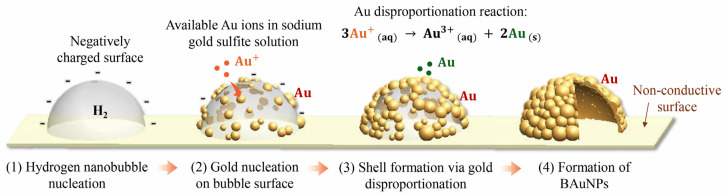
Schematic illustration of the formation mechanism of an individual BAuNP. A hydrogen nanobubble nucleates on the non-conductive surface, serving as a sacrificial template for the deposition of gold ions (orange color) via negative charge. The nucleation of gold (dark red color) at the gas–liquid interface subsequently attracts additional disproportionation gold atoms (green color), forming a continuous polycrystalline gold shell, resulting in BAuNP.

As illustrated in Figure 5, the synthesis of BAuNPs was initiated when the applied potential surpassed the hydrogen evolution reaction (HER) threshold of −0.55 V (vs Ag/AgCl) [48]. The localized hydrogen supersaturation initiates heterogeneous nucleation of nanobubbles at the electrode. We hypothesize that any interface, whether conductive or non-conductive, can serve as a heterogeneous nucleation site because these surfaces are significantly preferred over the bulk liquid phase due to a lower energy barrier, thereby facilitating the formation of hemispherical nanobubbles. The nanobubble geometry is determined by the balance of gas/liquid/solid interfacial energies, following the modified Young equation [47].

While nanobubble formation on the cathode was suppressed by electrodeposition of metal ions, non-conductive surfaces provide the necessary environment for hydrogen nanobubbles to nucleate and grow beyond the critical radius into a stable hemispherical geometry. Previous literature has established that nanobubbles typically exhibit a negative zeta potential, ranging from −10 to −45 mV, primarily due to the continuous adsorption of hydroxide ions (OH^−^) from the surrounding medium [49,50,51]. The electrostatic charge at the gas–liquid interface is governed by the interplay of volume contribution, interfacial energy, and electrostatic potential, according to Henry’s law. This negative zeta potential stabilizes the hydrogen nanobubble either by preventing coalescence and electrostatically attracting monovalent gold cations (Au^+^) from the sodium gold sulfite precursor.

The deposition of gold ions initiates the formation of a metallic gold shell across the gas–liquid interface. Once the initial gold layer is established, the gold surface facilitates the disproportionation of gold clusters in solution, promoting continuous, self-catalyzed growth via an electroless deposition mechanism and forming a porous shell. Although these nanobubbles are transient and difficult to observe directly, the size and geometry of nanobubbles are accurately evidenced by the resulting open core morphologies of BAuNPs.

### 3.1. Stripe-Patterned Electrode in an Electrochemical Bath

**Figure 6:**
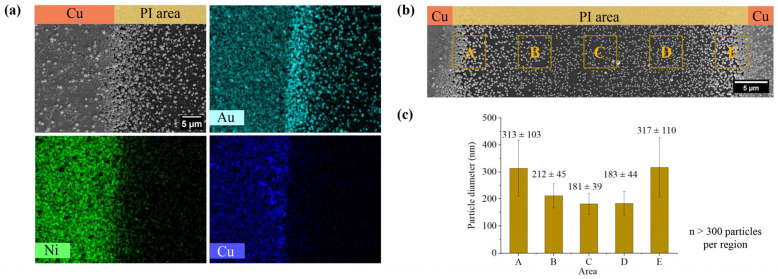
Characterization and spatial analysis of synthesized nanoparticles, (**a**) EDS elemental mapping illustrating the spatial distribution of Au, Ni, and Cu across the substrate. (**b**) FE-SEM micrograph capturing the morphology and density of AuNPs distributed across the electrode gap. (**c**) Statistical analysis of particle size distribution derived from FE-SEM data in (**b**), presenting average particle diameters across five distinct regions (A–E); diameters were calculated using a circular approximation method, indicating spatial variation in nanoparticle size.

After applying a constant potential of −0.7 V for 60 s, the working electrode patterns were cleaned, dried, and characterized via FE-SEM. Consistent with classical nucleation theory and our proposed hypothesis, the insulating PI surface functions as a heterogeneous nucleation site for hydrogen nanobubbles, which subsequently template the deposition of a gold shell. Figure 6a, FE-SEM imaging and EDS mapping, reveal a continuous gold layer on the conductive copper electrode, and isolated gold nanoparticles (AuNPs) distributed across the insulating PI gaps. Notably, nickel (Ni) was observed specifically on the conductive copper regions, where its deposition serves to maintain the availability of gold ions within the bulk electrolyte.

To quantify the influence of the hydrogen supersaturation gradient, particle size distributions were measured across five distinct 5 × 5 µm^2^ regions, totaling over 300 particles per area for statistical reliability. These areas were defined along the 50 µm-wide PI gap between two copper-stripped electrodes, from left to right, as illustrated in Figure 6b. For each location, measurements were performed in three areas at different positions. ImageJ analysis demonstrated a clear spatial dependence: AuNP diameters were maximal near the electrode edge, at 313 ± 103 nm, decreased to a minimum at the PI midpoint, 181 ± 39 nm, and increased again as they approached the opposing electrode, 317 ± 110 nm.

This symmetrical gradient confirms that the hydrogen concentration profile significantly influences AuNP diameters, a finding consistent with previous literature [47]. Furthermore, the particle density follows a similar trend, peaking near the copper edges and reaching a minimum at the PI midpoint due to the localized hydrogen supersaturation gradient. At the electrode–PI interface, the high particle density results in the formation of contiguous aggregates of gold nanoparticles.

**Figure 7:**
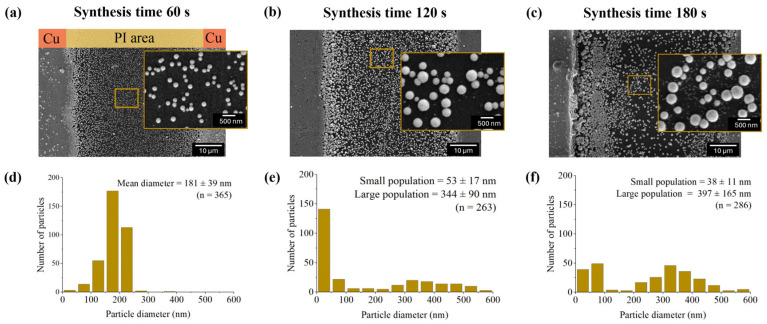
(**a**–**c**) FE-SEM micrographs and (**d**–**f**) corresponding size distribution histograms of AuNPs synthesized at varying durations: (**a**,**d**) 60 s, (**b**,**e**) 120 s, and (**c**,**f**) 180 s. Insets provide high-magnification views of the nanoparticle morphology. At a 60 s synthesis interval, the population has a mean diameter of 181 ± 39 nm (*n* = 365), whereas extended synthesis durations (120 s and 180 s) result in bimodal size distributions, indicating the simultaneous presence of both small and large particle populations.

Furthermore, as the synthesis duration was extended from 60 s to 120 s and 180 s, the mean particle diameter at the PI midpoint grew from 181 ± 39 nm (*n* = 365) to 344 ± 90 nm (*n* = 263) and 397 ± 165 nm (*n* = 286), respectively, as illustrated in Figure 7. This growth is attributed to the autocatalytic electroless deposition of gold onto pre-existing shells. However, the size distribution histograms for the 120 s and 180 s durations exhibit a bimodal distribution, indicating the emergence of a secondary population of smaller particles measuring approximately 53 ± 17 and 38 ± 11 nm, respectively. This prolonged synthesis increases the particle density near the edge of the copper electrode, effectively extending the active conductive surface area and accelerating the overall reaction rate. This expansion of the electrode also promotes rapid gold deposition, which prematurely encapsulates hydrogen nanobubbles and restricts their growth, resulting in the formation of small AuNPs observed in the highlighted regions of Figure 7b,c.

Consequently, synthesis under static conditions results in high polydispersity and poor structural definition, yielding submicron particles unsuitable for in vivo applications. Furthermore, the insufficient nanoparticle yield on the PI substrate under these conditions hindered the direct observation of the open-bowl structure. However, these results support the hypothesis that a planar, non-conducting surface facilitates the formation of hydrogen templates, which act as sacrificial templates for gold shell deposition. To overcome these limitations, a cyclonic flow system was designed to provide the shear force needed to detach particles from the PI surface while increasing uniformity by reducing localized supersaturation gradients. This strategy is intended to continuously regenerate active nucleation sites and maintain a homogeneous reaction environment, thereby enhancing the yield of individual nanoparticles.

**Figure 8:**
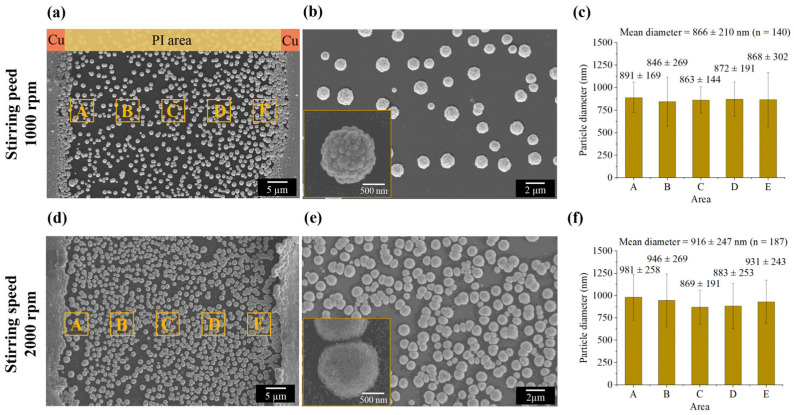
FE-SEM images and corresponding particle size distributions of AuNPs synthesized at varying stirrer speeds: (**a**–**c**) 1000 rpm and (**d**–**f**) 2000 rpm. The insets in (**b**,**e**) provide high-magnification views of the nanoparticle morphology. Panels (**c**,**f**) present statistical analyses of the size distributions derived from the images in (**a**,**d**), respectively. These charts illustrate the average particle diameters across five designated regions (A–E) within the electrode gap, calculated using a circular approximation method, and highlight the spatial variation in nanoparticle dimensions. Consequently, although macro-scale stirring enhances solution uniformity, it does not effectively produce BAuNPs smaller than 200 nm. To address these limitations, a microfluidic system was implemented to investigate the influence of dynamic forces and reduce aggregation through two primary strategies: (1) creating a unidirectional laminar flow to suppress random particle collisions and aggregation, and (2) precisely controlling reaction volumes and mass transport rates to limit particle growth within the target nanometric range.

Convective mass transport was generated using a DC motor-driven stirrer, employing cyclonic flow at 1000 and 2000 rpm to improve solution homogeneity and facilitate particle detachment from nucleation sites during the electrochemical process. FE-SEM images in Figure 8 show that the average particle size significantly increased to 866 ± 210 nm (*n* = 140) at 1000 rpm and 916 ± 247 nm (*n* = 187) at 2000 rpm after 180 s of synthesis. These results suggest that strong cyclonic forces lift growing particles from the PI substrate, while promoting inter-particle collisions and their subsequent incorporation into large microparticle aggregates.

Notably, the higher rotational speed of 2000 rpm yields microparticles with smoother surface morphologies than those synthesized at 1000 rpm, as highlighted in Figure 8b,e. This effect is likely due to increased shear forces at the surface interface, which disrupt the growth mechanisms of AuNPs by detaching them during the early stages of formation. This allows smaller particles to enter the bulk solution and eventually coalesce into smoother aggregated particles.

### 3.2. Electrochemical Microfluidic System

In accordance with classical nucleation theory, surface crevices serve as preferential nucleation sites by significantly reducing the activation energy required for bubble nucleation [52]. Gas molecules are attracted to these crevices, where they nucleate and subsequently grow into larger bubbles. This phenomenon is significantly influenced by surface wettability and local catalytic effects. When the interface of an entrapped gas pocket maintains a concave curvature [53], the bubble remains stable and tends to grow, even in an undersaturation environment. However, in a supersaturated surrounding liquid, the net gas flux toward the interface accelerates expansion, leading to a rapid increase in bubble diameter.

**Figure 9:**
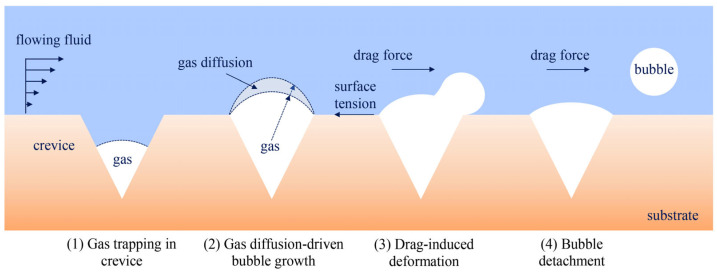
Schematic illustration of bubble nucleation from surface crevices under hydrodynamic flow. Gas is initially trapped within surface crevices, establishing stable nuclei. Dissolved gas diffuses from the bulk liquid into the nuclei, resulting in bubble growth. The dashed curved lines indicate the bubble interface/growth boundary, while the dashed blue arrows represent gas diffusion-driven bubble growth. Under fluid-flow conditions, hydrodynamic drag deforms the bubble, and detachment occurs when the hydrodynamic force exceeds the surface tension at the crevice interface.

As the growing bubble reaches the mouth of the crevice, external shear forces overcome surface tension, facilitating its detachment and the release of a free bubble with a gold shell into the solution. Following departure, a residual volume of gas remained trapped within the crevice, serving as a seed for the next formation cycle. Furthermore, convective flow enhances gas mass diffusion, thereby accelerating growth, detachment, and subsequent re-nucleation by ensuring a continuous supply of fresh, supersaturated solution to the interface. As illustrated in Figure 9, the process of nanobubble formation under shear flow is divided into four distinct stages: (1) a pocket nucleates within a crevice, (2) the bubble grows to the crevice edge, (3) it deforms due to shear force, and (4) it detaches from the nucleation site.

By integrating theoretical and experimental analyses, Groß and Pelz [52] investigated how this process is influenced by the shear rate ($\dot{\gamma }$), liquid supersaturation (ξ), and crevice diameter. They established that the reduced nucleation rate (f/ξ) follows a scaling law of the form $f/\xi \propto {\dot{\gamma }}^{\alpha }$, where the nucleation rate (f) is defined as the ratio of the diffusion mass flux ($\dot{m}$) to the mass of the detaching bubbles ($m_{B}$). Within this model, the exponent α serves as a non-linearity index, quantifying the degree to which the nucleation kinetics deviate from a linear response to the applied shear. Furthermore, the wall shear rate at the channel surface is governed by the flow rate(Q) and the channel dimensions (h and w), following $\dot{\gamma }=6Q/h^{2}w$. The corresponding wall shear stress (τ) can then be estimated from the fluid dynamic viscosity (μ) using $\tau =\mu \dot{\gamma }$. The existence of shear stress, which is directly proportional to the fluidic drag force, is the primary mechanism for releasing particles from the electrode surface. Particle detachment occurs at the critical point where the hydrodynamic shear stress exerted by the fluid exceeds the adhesion energy between the templated nanobubble and the nucleation site.

Drawing upon the described bubble-formation phenomena, the formation of the gold shell begins with the growth of a bubble. As the bubble grows, its surface develops a negative charge density that is sufficient to attract gold ions from the electrolyte. This attraction leads to the deposition of gold on the bubble surface, forming an open, hemispherical gold structure shaped by the gas–liquid interface. Once the gold-coated bubble reaches the edge of the nucleation site, it becomes susceptible to external shear forces. The resulting drag force eventually overcomes the adhesive forces, facilitating detachment and lifting both the bubble and its gold shell into the bulk electrolyte. Accordingly, the flow rate directly influences the kinetics of nucleation, growth, and detachment. This indicates that the flow rate is defined as a critical synthesis parameter that is systematically evaluated in the final section of this study.

#### 3.2.1. Channel Length

To investigate the synthesis yield of BAuNPs, a microfluidic platform integrated with an electrochemical configuration, consisting of a single cathode between dual anodes, was employed. This setup was designed to modulate reaction kinetics and suppress particle aggregation by precisely confining the reaction volume. A constant voltage of −0.7 V was applied to the cathode, while a custom-built syringe pump maintained a constant flow rate of 0.135 µL s^−1^. To evaluate the influence of reaction time on nanoparticle yield and morphology, 0.5 mm-high, 1.4 mm-wide microfluidic channels with two different active electrode pathways, 7.5 mm and 15.0 mm, measured from the electrode edge to the outlet, were employed.

**Figure 10:**
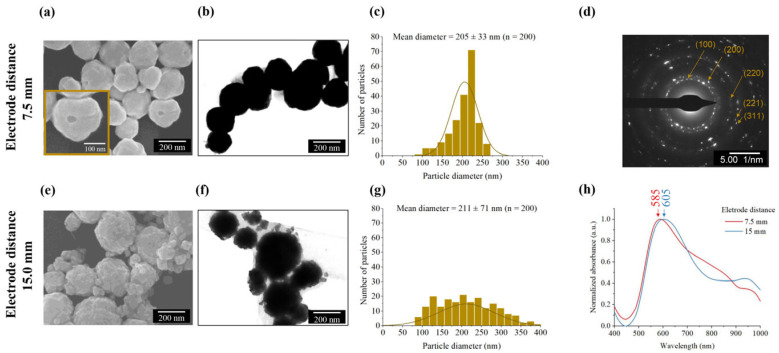
Characterization of the morphology, particle size distributions, crystal structure, and optical properties of AuNPs synthesized via the electrochemical microfluidic system at varying channel length. (**a**–**c**) FE-SEM image, TEM image, and particle size distribution of AuNPs synthesized using a 7.5 mm channel. The yellow box in (**a**) shows the open structure of the particle. (**d**) SAED pattern confirming the polycrystalline nature of the synthesized AuNPs. (**e**–**g**) FE-SEM image, TEM image, and particle size distribution of AuNPs synthesized using a 15.0 mm channel. (**h**) Normalized UV–Vis absorption spectra comparing the AuNPs synthesized under both conditions.

FE-SEM analysis revealed that particles synthesized within the 7.5 mm channel exhibited the desired open-shell structure, as highlighted in Figure 10a, confirming the successful utilization of hydrogen nanobubbles as sacrificial templates. Conversely, this morphology was absent in particles from the 15.0 mm channel due to particle aggregation during excessive flow, as shown in Figure 10e. Interestingly, the average diameters of BAuNPs from both the 7.5 mm and 15.0 mm channels were similar, measuring approximately 205 ± 33 nm (*n* = 200) and 211 ± 71 nm (*n* = 200), respectively. The extended residence time in the longer channel facilitated secondary particle–particle collisions, leading to the observed aggregation.

Even though the open-shell structures in both conditions cannot be clarified by TEM imaging due to the relatively small opening size and the dense, thick shell, the corresponding selected-area electron diffraction (SAED) pattern exhibits multiple diffraction rings indexed to the (111), (200), (220), and (311) planes, confirming the polycrystalline nature of the synthesized gold [54]. The absorption spectra from both channel lengths exhibited similar resonance peaks at approximately 585 nm and 605 nm. These findings indicate the optical characteristics of aggregated nanoparticles, aligning with previous studies [55,56]. It was mentioned that individual gold nanoparticles typically exhibit a resonance peak around 520 nm and shift to longer wavelengths, 620 nm, when they aggregate or reach diameters of approximately 200 nm.

**Figure 11:**
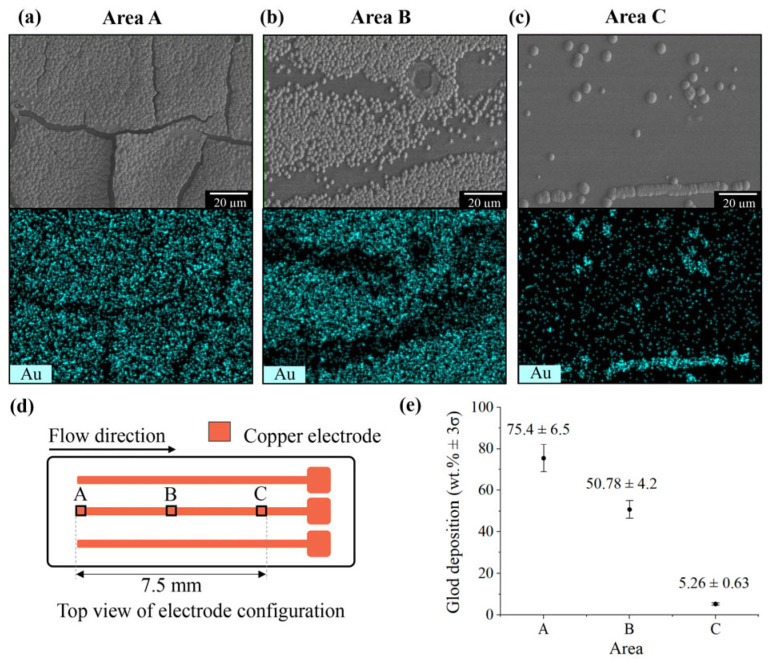
EDS mapping and quantitative analysis of gold deposition along the 7.5 mm electrode length. (**a**–**c**) SEM images and corresponding EDS elemental maps (analysis area = 96 × 123 µm^2^) illustrating the gold distribution (cyan color) at regions A, B, and C, respectively. (**d**) Schematic representation of the microfluidic channel, indicating the flow direction and the specific measurement positions along the electrode. (**e**) Quantitative comparison of gold deposition (wt% ± 3σ) across the designated regions, demonstrating a significant decrease in gold deposition efficiency along the flow pathway. Error bars represent ± 3σ.

To optimize the channel length for BAuNPs synthesis and minimize particle aggregation, the axial reaction distribution was characterized using EDS mapping. As illustrated in Figure 11, the 7.5 mm conductive path was divided into three distinct regions: A, B, and C. EDS mapping revealed a sharp decline in gold deposition efficiency along the channel, with gold concentrations dropping from 75.4 ± 6.5 wt% at position A to 50.78 ± 4.2 wt% at position B, and further to 5.26 ± 0.63 wt% at position C. The minimal gold percentage at position C suggests that the gold ions in electrolyte become insufficient to support a significant electrochemical reaction, even for electrodeposition. Consequently, extending the channel length beyond 7.5 mm does not increase the size or generate additional particles; this explains why the average diameters for 7.5 mm (205 ± 33 nm) and 15.0 mm (211 ± 71 nm) were statistically similar. This conclusion is further supported by the observation that the average current density of both channel lengths was similar, approximately 5.5 mA cm^−2^. Based on these results, a 7.5 mm microchannel was selected as the optimal length to minimize aggregation while ensuring sufficient gold deposition.

Despite the improvements afforded by the 7.5 mm channel, the synthesized particles remained slightly above the 200 nm threshold, suggesting that the initial reaction kinetics were excessively rapid. We hypothesize that high deposition rates prematurely encapsulate hydrogen nanobubbles, preventing their growth related to the size of the open-shell cavity. To address this, subsequent experiments will prioritize reducing the applied potential and the flow rate. A decrease in applied potential is expected to slow both the hydrogen nucleation rate and the gold reduction kinetics, providing sufficient time for nanobubble growth. Simultaneously, modulating the flow rate will allow optimization of the surface drag force, which, at excessive flow rates, can prematurely detach the hydrogen templates and promote undesirable particle aggregation.

#### 3.2.2. Applied Voltage

**Figure 12:**
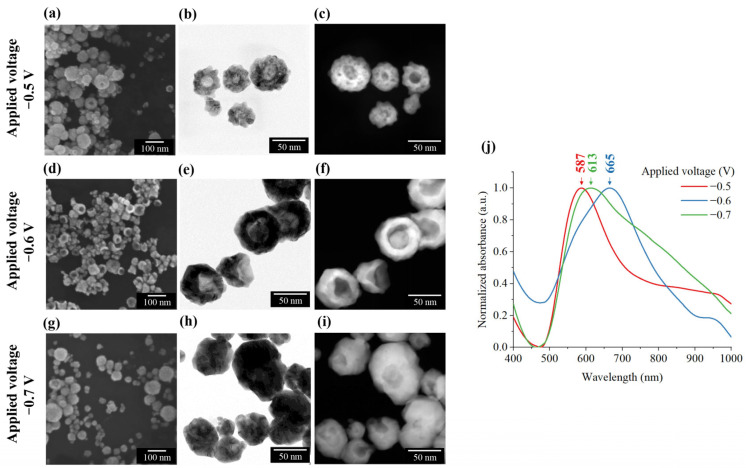
Morphology, optical, and electrochemical behavior of BAuNPs synthesized under varying applied voltages. FE-SEM images, HR-TEM images, and STEM images of BAuNPs synthesized under (**a**–**c**) −0.5 V, (**d**–**f**) −0.6 V, and (**g**–**i**) −0.7 V applied potentials. (**j**) Normalized UV–Vis absorbance of BAuNPs colloidal from various applied voltages.

To refine the morphology of the synthesized BAuNPs, the applied potential was systematically reduced from −0.7 V to −0.5 V. During these experiments, the flow rate was maintained at 0.028 µL s^−1^, approximately five times lower than the rate used in previous experiments. Figure 12 confirms the successful formation of open-bowl structures across all applied potentials, with core diameters ranging from 15 nm to 40 nm and total particle sizes between 30 nm and 100 nm. The reduction in flow rate effectively decelerates the gold deposition kinetics, providing sufficient time for the hydrogen nanobubble templates to grow before encapsulation.

At a relatively low potential of −0.5 V, SEM, TEM, and STEM images reveal a small cavity enclosed with a hemispherical porous shell, as shown in Figure 12a–c. This structure is attributed to a slow reaction rate that provides an insufficient hydrogen supply, yielding smaller bubbles. This low reaction rate also leads to an undersupply of gold, allowing secondary hydrogen nanobubbles to disrupt shell formation by heterogeneously nucleating on the pre-existing gold layer, resulting in a porous shell. Consequently, the UV–Vis spectrophotometer shows an absorbance peak at 587 nm, which closely aligns with the optical signatures of conventional hollow gold nanoparticles.

In contrast, synthesis at −0.6 V yields individual BAuNPs with well-defined, solid gold shells and core diameters of 30–40 nm. This morphological transition induces a redshift in the absorption peak from 587 to 665 nm, indicating a more pronounced cavity. This peak position represents a significant redshift, compared with the theoretical absorbance values for solid gold nanospheres (540 nm) and hollow gold nanoparticles (564 nm) with the same outer diameter, calculated using full Mie theory, which serves as a baseline reference for idealized spherical geometries [57,58,59], as mentioned in Supplementary Material S1. A non-uniform population characterized by significantly smaller cores with thick, solid shells was defined when the potential was further increased to −0.7 V. Under these conditions, the high gold deposition rate prematurely encapsulates the hydrogen bubbles, capturing their growth. This structural transformation causes the absorption peak to blue-shift toward 613 nm.

Previous studies have reported that BAuNPs exhibit broad optical absorption bands within the 550–700 nm range [32,60], which is in good qualitative agreement with the experimental UV–Vis peaks observed in this study. These findings demonstrate that the planar E-MFC surface can produce individual BAuNPs with high uniformity and dimensions well below 200 nm, which is required for in vivo biomedical applications. Based on these results, an applied potential of −0.6 V was selected as the optimal value for subsequent flow rate optimization studies.

#### 3.2.3. Flow Rate

**Figure 13:**
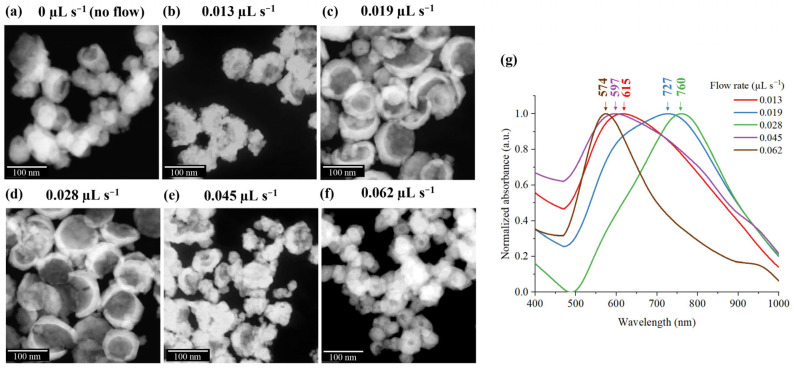
Morphological characterization and absorbance spectra of BAuNPs synthesized at various flow rates within the electrochemical microfluidic system. FE-SEM micrographs illustrating the resulting nanoparticle morphologies under (**a**) a static (no-flow) condition compared to (**b**–**f**) flow rates of 0.013, 0.019, 0.028, 0.045, and 0.062 µL s^−1^, respectively. (**g**) Normalized UV–Vis absorbance spectra of the BAuNPs synthesized across the various flow rates, demonstrating the influence of hydrodynamic conditions on the LSPR properties.

The continuous influx of fresh precursor solution significantly modulates the competitive interplay between hydrogen nanobubble growth and gold shell deposition. To evaluate this relationship, flow rates were varied from 0.013 µL s^−1^ to 0.062 µL s^−1^ and compared with a static (no-flow) condition. Under static conditions, the STEM image in Figure 13a indicates poorly defined core structures due to the absence of a fresh solution. The localized hydrogen concentration is inadequate to nucleate and enlarge nanobubbles before gold deposition begins. Consequently, the resulting low yield was insufficient for UV–Vis Spectrophotometric characterization. 

The low influx of fresh precursor at 0.013 µL s^−1^ increases localized hydrogen concentrations, thereby improving core visibility, as shown in Figure 13b. The resulting particles, associated with a calculated wall shear stress of 0.223 mPa, exhibit an average core size of 47.5 ± 14.0 nm, an outer diameter of 77.6 ± 16.6 nm, and an absorbance peak at 615 nm. However, a porous gold shell morphology is observed because the undersupply of gold ions prevents the formation of a continuous, solid polycrystalline gold shell before secondary hydrogen nanobubbles nucleate heterogeneously on the pre-existing gold shell surface.

Increasing the flow rate and wall shear stress to 0.019 µL s^−1^ and 0.326 mPa promotes an enlargement of the core structure to 74.6 ± 13.8 nm, encapsulated by a solid gold shell, resulting in an average particle diameter of 102.7 ± 15.2 nm, as indicated by the TEM image in Figure 13c. This morphology successfully redshifts the absorption peak further to 727 nm. However, a bimodal size distribution is observed, as the relatively low convective shear force is insufficient to completely eliminate localized ion concentration gradients. This heterogeneity in particle size consequently manifests as a broadened absorbance spectrum.

The optimal configuration was achieved at a flow rate of 0.028 µL s^−1^. At this velocity, the increased influx of precursor accelerates reaction kinetics, facilitating the growth of large, uniform core structures with an average diameter of approximately 80.1 ± 11.9 nm and well-defined solid shells. The increase in wall shear stress at 0.480 mPa yields highly uniform BAuNPs with an increasing total diameter to 105.3 ± 12.1 nm, as presented in Figure 13d. Consequently, the absorbance peak redshifts to 760 nm, effectively aligning the optical response with the biological window. The high structural and dimensional uniformity of these BAuNPs results in a significantly narrowed absorbance spectrum, which is advantageous for precise sensing and imaging applications.

Conversely, exceeding the optimal flow rates of 0.045 µL s^−1^ and 0.062 µL s^−1^ adversely affects the open-bowl geometry. A continuously higher flow rate increases the drag force at the surface, resulting in higher wall shear stresses of 0.771 mPa and 1.063 mPa, respectively, which induce premature bubble detachment from the nucleation sites. This shortened growth period, combined with an elevated gold deposition rate, limits bubble enlargement, resulting in small cores with thick shells. At 0.045 µL s^−1^, the average core diameter decreases to 46.1 ± 14.1 nm, and the outer diameter is around 73.2 ± 16.8 nm, as shown in Figure 13e. At 0.062 µL s^−1^, the core diameter drops sharply to 18.4 ± 5.0 nm with a total diameter of 45.3 ± 10.2 nm, as shown in Figure 13f. These structural changes cause the blue shifts in the absorption spectra toward 597 nm and 574 nm, respectively. These findings indicate that the optical characteristics of BAuNPs are also highly sensitive to the maximum core-to-particle diameter ratio, as shown in Table 1, which is dictated by the critical balance between localized ion concentration and surface shear forces.

The study demonstrates that flow rate is a critical determinant of core structure and particle uniformity, as it modulates the equilibrium between chemical reaction rates and surface drag forces. By utilizing an electrochemical microfluidic system at an applied potential of −0.6 V and an optimal flow rate of 0.028 µL s^−1^, the synthesis process successfully generates individual BAuNPs with a uniform diameter of 105.3 ± 12.1 nm and an absorption peak at 760 nm. Given their precise size profile and strong NIR activity within the biological window, these BAuNPs represent a promising, biocompatible candidate for in vivo applications. Additionally, after circulating through the bloodstream, nanoparticles with a diameter of 100 nm will predominantly accumulate in the liver and spleen, where they are typically cleared by the mononuclear phagocyte system (MPS), which serves as the body’s natural mechanism for bloodstream clearance [61]. To evaluate the commercial and clinical viability of this nanomaterial, the production yield under optimized synthesis conditions was quantified. Analysis of representative samples, 3 × 1 mL of the BAuNPs colloidal, via dynamic light scattering (DLS), revealed an average particle concentration of approximately 1.36 × 10^8^ particles mL^−1^. Relying on the bulk density of gold and ideal geometric approximations detailed in Supplementary Section S2 [62], the estimated mass concentration of the BAuNP suspension is approximately 0.67 µg mL^−1^. However, the synthesis-dependent formation of polycrystalline shells introduces variations in surface roughness and porosity. These morphological factors may introduce deviation between the estimated and actual mass concentration, as the calculations are based on an idealized hemispherical shell approximation. Production yields can be significantly scaled by integrating parallelized electrode arrays under expanded microfluidic channel dimensions. Furthermore, the parameters established in this work provide baseline information for future optimization of pH, volume, and chemical concentrations to further refine the optical properties and structural uniformity of the particles.

## 4. Conclusions

Preliminary synthesis of BAuNPs using copper stripe patterns in an electrochemical bath demonstrated that static conditions yield non-uniform, sub-micron particles across non-conductive surfaces. Although the introduction of cyclonic flow improves particle uniformity by reducing supersaturation gradients, it simultaneously promotes microparticle aggregation, thereby obscuring the discrete open-bowl structure. To overcome this limitation, an E-MFC utilizing laminar flow was implemented to produce uniform, individual BAuNPs with dimensions below 200 nm. In this configuration, microchannel length remains a critical design parameter, as excessive travel distance promotes particle–particle collisions and subsequent aggregation rather than increasing the overall yield.

Our findings indicate that the structure and morphology of BAuNPs are significantly influenced by the kinetic interplay between the growth rate of hydrogen nanobubbles and the deposition rate of the gold shell. Even though these mechanisms are primarily regulated by the applied potential, the continuous influx of fresh electrolytes enables precise tuning of their formation. Additionally, the flow rate dictates the wall shear stress within the microfluidic system. An optimized flow rate homogenizes ion concentrations across the supersaturation gradient and facilitates particle detachment from nucleation sites, without undesirable particle accumulation during synthesis.

Achieving a synergy between these electrochemical and hydrodynamic parameters enables controlled bubble growth and precise timing of gold deposition, producing well-defined open-bowl structures with thin, solid gold shells. This structural optimization effectively shifts the LSPR peak of the BAuNPs into the biological window. Consequently, the development of controllable synthesis strategies for colloidal BAuNPs and the exploration of their structure-dependent plasmonic behavior represent critical advancements for biomedical applications.

## Figures and Tables

**Table 1:** Structural dimensions and calculated wall shear stress of bowl-shaped gold nanoparticles (BAuNPs) synthesized under varying flow rate conditions.

Flow Rate (µL s^−1^)	Wall Shear Stress (mPa)	Particle Diameter (nm)		Core Diameter (nm)		Shell Thickness (nm)		Core/Particle Ratio	
		Mean	SD	Mean	SD	Mean	SD	Mean	SD
0	0	59.1	16.9	56.1	14.4	N/A	N/A	N/A	N/A
0.013	0.223	77.6	16.6	47.5	14.0	15.0	2.7	0.61	0.09
0.019	0.326	102.7	15.2	74.6	13.8	14.1	2.7	0.73	0.06
0.028	0.480	105.3	12.1	80.1	11.9	12.6	1.9	0.76	0.04
0.045	0.771	73.2	16.8	46.1	14.1	13.5	3.0	0.62	0.09
0.062	1.063	45.3	10.2	18.4	5.0	13.5	3.5	0.41	0.06

Note: N/A = Not applicable. Particle size data (mean ± SD) were calculated by aggregating measurements from three independent synthesis batches (*n* > 60 particles). Calculations were performed assuming a dynamic viscosity (μ) of 0.001 Pa·s (water at room temperature).

## Data Availability

The original contributions presented in this study are included in the article/Supplementary Material, further inquiries can be directed to the corresponding author.

## Supplementary Materials

The following supporting information can be downloaded at: https://www.mdpi.com/article/10.3390/nano16100640/s1.

## Abbreviations

The following abbreviations are used in this manuscript:

BAuNPs	Bowl-shaped gold nanoparticles
E-MFC	Electrochemical microfluidic chip
LSPR	localized surface plasmon resonance
