# Supplementary material for: Enhancing the Uniformity of Bowl-Shaped Gold Nanoparticles Using a Dynamic System in an Electrochemical Microfluidic Chip
Source: Nanomaterials (Basel). 2026 May 21;16(10):640. doi: 10.3390/nano16100640 (PMC13209651; doi:10.3390/nano16100640)
Supplement: Supplementary file 1 [file nanomaterials-16-00640-s001.zip › nanomaterials-4297279-supplementary.pdf]

Supplementary Materials

S1. Numerical analysis of electromagnetic properties

To evaluate the influence of the bowl-shaped geometry on plasmonic behavior, numerical calculations were performed using particle dimensions obtained from STEM analysis, an outer diameter of 60 nm, and a core diameter of 35 nm. For comparison, a solid gold nanosphere (AuNPs) with a diameter of 60 nm and a concentric hollow gold nanosphere (HAuNPs) with an outer diameter of 60 nm and a core diameter of 35 nm were modeled using full Mie theory, described by Bohren and Huffman [57]. Calculations were performed using the nanoComposix Mie Theory Calculator [59], and the numerical solution developed by Steven J. Oldenburg [58] for concentric core–shell nanoparticles, with standard optical constants for gold in an aqueous medium with a refractive index of 1.33.

The extinction cross-section (C_{ext}) is expressed as

$$C_{ext} = \left(\frac{2\pi}{k^2}\right)\sum_{n=1}^{\infty}(2n+1)\,Re(a_n + b_n) \quad \text{Eq. (S1)}$$

where k is the wave number and a_n and b_n are the Mie coefficients derived from Riccati–Bessel functions [a2]

$$a_n = \frac{m\psi_n(mx)\psi_n'(x) - \psi_n(x)\psi_n'(mx)}{m\psi_n(mx)\xi_n'(x) - \xi_n(x)\psi_n'(mx)} \quad \text{Eq. (S2)}$$

$$b_n = \frac{\psi_n(mx)\psi_n'(x) - m\psi_n(x)\psi_n'(mx)}{\psi_n(mx)\xi_n'(x) - m\xi_n(x)\psi_n'(mx)} \quad \text{Eq. (S3)}$$

where m is the relative refractive index, x is the size parameter, and ψ_n and ξ_n are the Riccati–Bessel functions defined as

$$\psi_n(x) = xj_n(x) \quad \text{Eq. (S4)}$$

$$\xi_n(x) = xh_n^{(1)}(x) \quad \text{Eq. (S5)}$$

where $j_n(x)$ is the spherical Bessel function and $h_n^{(1)}(x)$ is the spherical Hankel function of the first kind.

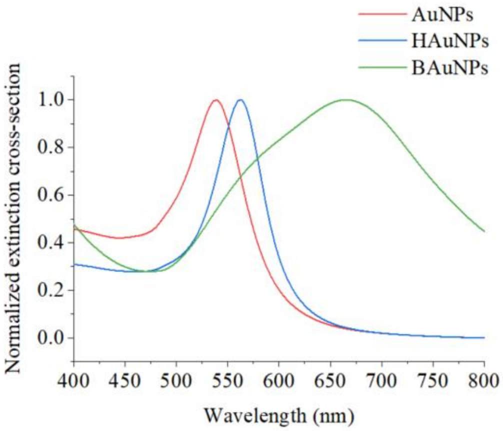

Figure S1. Normalized extinction cross-sections of 60 nm solid gold nanospheres exhibit a peak at 540 nm, and hollow gold nanospheres with 60 nm outer diameter and 35 nm cavity show a redshift to 564 nm. Notably, the experimental BAuNPs exhibited a pronounced redshift to 665 nm, highlighting the impact of the open-bowl structure.

The calculated extinction spectra are presented in Figure S1. Solid AuNPs with a 60 nm diameter exhibit a surface plasmon resonance (SPR) peak at 540 nm, while HAuNPs with an outer diameter of 60 nm and a core diameter of 35 nm show a red-shifted SPR peak at approximately 564 nm. Notably, the experimentally synthesized bowl-shaped gold nanoparticles (BAuNPs) exhibit a more significant redshift, with an SPR peak at approximately 665 nm. This shift demonstrates the strong influence of the open-shell bowl geometry on the plasmonic response compared to both solid and closed hollow spherical geometries.

S2. Yield estimation of bowl-shaped gold nanoparticles

The mass concentration (C_m) of BAuNPs was estimated by combining the particle number concentration (N = 1.36 × 10^8 particles mL^{-1}) from dynamic light scattering (DLS), with the calculated mass of a single nanoparticle ($m_{particle}$):

$$C_m = N \times m_{particle} \quad \text{Eq. (S6)}$$

$$m_{particle} = \rho V_{bowl} \quad \text{Eq. (S7)}$$

$$V_{bowl} = \frac{2}{3}\pi(R_o^3 - R_i^3) \quad \text{Eq. (S8)}$$

Using the bulk density of gold (ρ = 19.32 g cm^{-3}) [61]. A calculated geometry was assumed as an ideal open structure with a hemispherical shell. The volume in this calculation was approximated from synthesized BAuNPs from the optimal condition (the average particle diameter of 105.3 nm with a core diameter of 80.1 nm, and shell thickness of 12.6 nm). Accordingly, the mass concentration was estimated to be 0.67 μg mL^{-1}, when R_o = 52.65 nm and R_i = 40.05 nm.
